# A Case Series of Elizabethkingia meningosepticum Bacteremia in the Cancer Population

**DOI:** 10.7759/cureus.18627

**Published:** 2021-10-09

**Authors:** Dae Hyun Lee, Raj H Patel, Ishita Mehra, Rahul Shenoy, Sowmya Nanjappa, John Greene

**Affiliations:** 1 Cardiology, University of South Florida, Tampa, USA; 2 Internal Medicine, Edward Via College of Osteopathic Medicine, Monroe, USA; 3 Internal Medicine, North Alabama Medical Center, Florence, USA; 4 Internal Medicine, Case Western Reserve University, Cleveland, USA; 5 Infectious Disease, Moffitt Cancer Center, Tampa, USA; 6 Internal Medicine, Moffitt Cancer Center, Tampa, USA

**Keywords:** cancer patients, severe sepsis, neutropenia, urinary tract infection, gram-negative bacteremia, coagulase-negative staphylococcus, elizabethkingia

## Abstract

*Elizabethkingia meningosepticum* (*E.* *meningosepticum*) is a ubiquitous microorganism previously known as *Chryseobacterium meningosepticum. *It is emerging as a pathogen responsible for bacteremia in the immunocompromised population, particularly in cancer patients and those with a history of prolonged hospital stay and frequent instrumentations. A retrospective chart review of all cases over 10 years at the Moffitt Cancer Center showed a total of three patients with *E.* *meningosepticum* infection. The first patient (history of multiple myeloma) underwent endoscopy complicated by aspiration pneumonia and positive blood culture for *E.* *meningosepticum* infection. The second patient (current acute myelogenous leukemia) had neutropenic fever in the setting of a recent chest port infection. Blood culture from the chest port showed *E. meningosepticum*. The third patient (history of esophageal adenocarcinoma and acute myelogenous leukemia) had a history of recent pneumonia and cellulitis who presented with recurrent neutropenic fever. Blood culture was positive for *E. meningosepticum*. *E.* *meningosepticum* bacteremia has a high 28-day mortality rate (41%). As these three cases illustrate, early identification of the pathogen along with empiric treatment with a fluoroquinolone and/or minocycline is indicated to reduce morbidity and mortality.

## Introduction

*Elizabethkingia* is ubiquitously found in nature and is not a part of the normal human flora [1]. It is a biofilm-forming, non-motile, non-fermenting beta-hemolytic, oxidase and catalase-positive, Gram-negative bacilli that grow yellow-colored colonies after 48 hours of incubation at 37° C in enriched media. *Elizabethkingia meningosepticum* (*E. meningosepticum*) was originally identified as *Flavobacterium* *meningosepticum* by Elizabeth King in 1959 as she studied unclassified bacteria associated with meningitis in infants [2]. The genus was reclassified in 1994 and the bacteria with the aforementioned characteristics were placed under *Chryseobacterium meningosepticum*. By 2005, after extensive phylogenetic studies, it was placed under the genus *Elizabethkingia* [1].

*Elizabethkingia* infections were more commonly associated with outbreaks of neonatal meningitis; however, they have recently emerged to be an important cause of bacteremia in immunocompromised individuals, especially in those with a history of prolonged hospital stays and frequent instrumentation [3,4]. *E. meningosepticum* survives in chlorine-treated municipal water and colonizes sinks, basins, and saline solutions. It also has the propensity to colonize medical equipment, making it a significant cause of nosocomial infections in immunocompromised individuals [5]. In this case series, we report three cases of *E. meningosepticum* bacteremia seen in patients with underlying malignancies who were successfully treated with a multidrug regimen.

## Case presentation

Case 1

**Figure 1:**
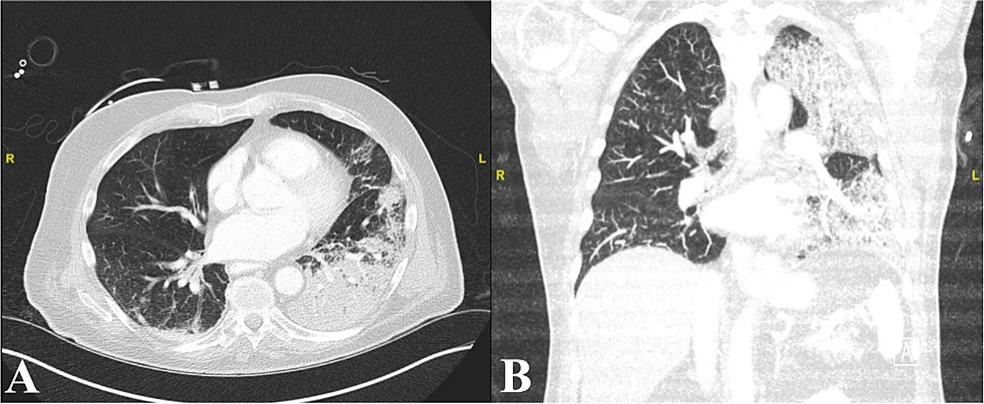
CT of the thorax on axial (A) and coronal (B) planes showing extensive consolidation of the left lung consistent with multifocal pneumonia

A 72-year-old male with a past history of metastatic melanoma (treated with excision and radiation, with no current evidence of recurrence), Barrett’s esophagus, and asthma presented for a routine follow-up endoscopy for Barrett’s esophagus. During the endoscopy, the patient became hypoxic after induction of anesthesia and had wheezing. The patient was intubated and admitted to the ICU for possible bronchospasm or aspiration. Vital signs were significant for hypotension (90/69 mmHg), tachycardia (139 bpm [beats per minute]), and temperature of 35.9° C. Labs were notable for leukocytosis (11,000/ml), pre-renal acute kidney injury (blood urea nitrogen (BUN) 21 mg/dL, creatinine 1.4 mg/dL), and respiratory acidosis (pH 7.22, pCO2 60.0). CT of the thorax, abdomen, and pelvis showed extensive consolidation predominantly in the left lung and the right lower lobe consistent with multifocal pneumonia (Figure 1). Meropenem and minocycline were empirically started. Blood cultures were positive for *E. meningosepticum* and susceptible to ciprofloxacin and minocycline. Antibiotics were changed to ciprofloxacin, cefoxitin, minocycline, and metronidazole for broad-spectrum coverage until the susceptible organism was identified. Following the identification of *E. meningosepticum*, antibiotic coverage was deescalated to align with susceptibility results. His respiratory status improved, and he was extubated after 48 hours. Follow-up blood culture had no growth. He continued to improve clinically and was discharged on ciprofloxacin and minocycline for an additional 10 days.

Case 2

A 50-year-old female with relapsed acute myelogenous leukemia (AML), previously treated with 7+3 induction therapy and high-dose cytarabine cycle for consolidation was admitted for cladribine-based salvage chemotherapy. She was recently hospitalized at another institute for a chest port infection. She was treated with intravenous vancomycin, but the port was not removed. At the time of admission, she was febrile (38.5° C), normotensive (118/73 mmHg), had tachycardic (119 bpm), and had a normal respiratory rate. Labs were notable for bicytopenia (hemoglobin 9.7 g/dL, platelets 26,000/ml) and a leukocyte count of 4,050/mL. She was started on empiric antibiotics with meropenem, minocycline, and tobramycin with the removal of the chest port. Blood cultures were positive for *Leclercia adecarboxylata* and *E. meningosepticum* (susceptible to ciprofloxacin and minocycline). She was transitioned to ciprofloxacin, meropenem, and minocycline. She later developed neutropenic fever for which voriconazole and acyclovir were added. Repeat blood culture was negative and she was discharged to hospice after relapsed leukemia was noted per bone marrow biopsy after the initiation of cladribine-based salvage chemotherapy.

Case 3

**Figure 2:**
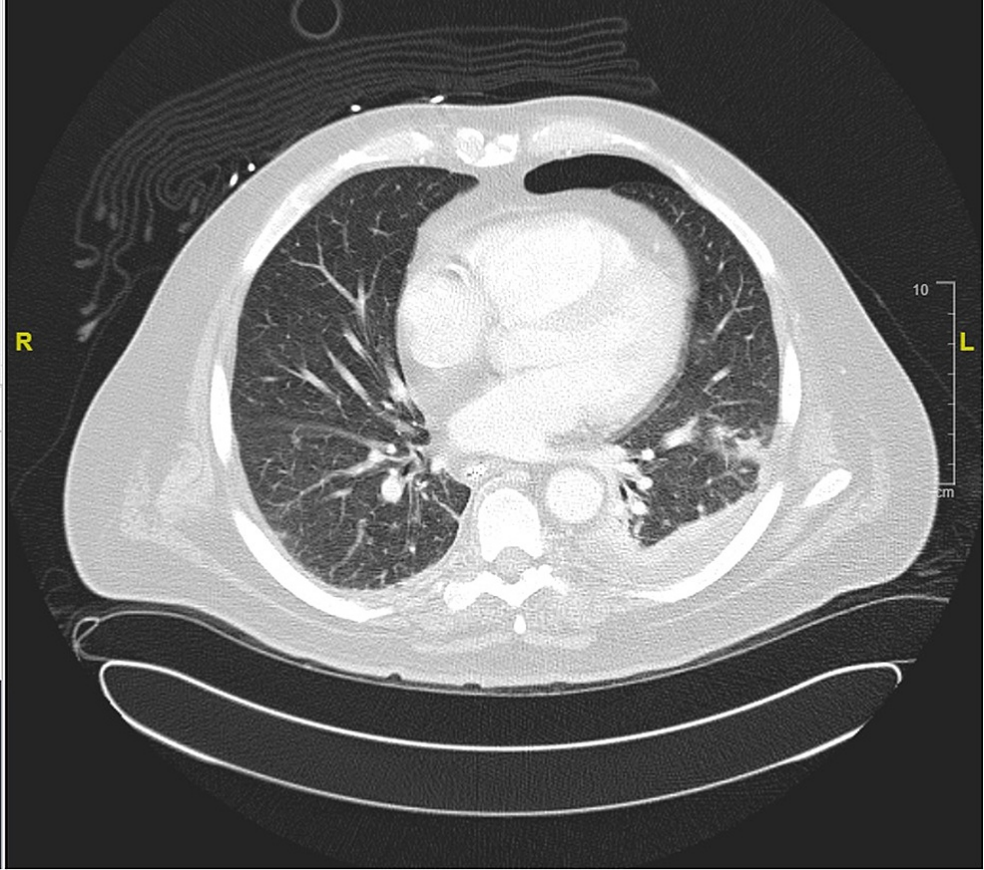
CT of the thorax showing regions of pulmonary consolidation in the left lung

A 65-year-old male with an oncologic history of adenocarcinoma of the esophagus (stage IV) and AML was treated with 5-azacytidine (Vidaza®). He was admitted with a new onset of productive cough with blood-tinged sputum for two days and pleuritic chest pain. His past infectious history was notable for pneumonia caused by *Pseudomonas aeruginosa* followed by mild pneumonia and right lower extremity cellulitis. He recently completed a treatment course of daptomycin. On examination, he was normotensive (BP 140/70 mmHg), had tachycardia (110 bpm), and had a normal respiratory rate with an oxygen saturation of 97% on room air. Physical examination was notable for decreased breath sounds and a right upper chest port with no evidence of infection. His labs were significant for pancytopenia (WBCs: 2,200/mL, absolute neutrophil count (ANC): 1010/mL, hemoglobin: 8.7 g/dL, platelets: 13,000/mL), and acute kidney injury (AKI) (creatinine: 1.4 mg/dL). CT of the thorax showed clustered ground glass nodularity in the left lower lobe indicating focal infection, pulmonary consolidation and adjacent atelectasis of the left lower lobe, and a small left pneumothorax (Figure 2). He was empirically started on piperacillin-tazobactam, which was later changed to meropenem after isolation of Gram-negative rods from blood cultures. Blood and sputum cultures were positive for *E. meningosepticum* sensitive to minocycline, and ciprofloxacin and minocycline were initiated (Table 1). Follow-up blood cultures were negative. His hospital course was complicated by multi-organ failure (worsening AKI, septic shock, and acute respiratory failure) and required tracheostomy. Following goals of care discussion, withdrawal of care was decided and comfort measures were initiated. He subsequently expired two days later.

**Table 1:** Susceptibility results for Elizabethkingia meningosepticum in Case 3 R: resistant; I: intermediate; MDIL: minimum dilutional inhibitory level; MINT: minimum inhibitory concentration interpretation

Antibiotic	MDIL	MINT
Ampicillin	≥32	R
Ampicillin/Sulbactam	≥​​​​​​​32	R
Cefazolin	≥​​​​​​​64	R
Cefepime	32	R
Cefoxitin	≥​​​​​​​64	R
Ciprofloxacin	2	I
Gentamicin	≥​​​​​​​16	R
Meropenem	≥​​​​​​​16	R
Piperacillin/Tazobactam	≥​​​​​​​128	R
Tobramycin	≥​​​​​​​16	R
Trimethoprim/Sulfa	80	R

## Discussion

Infection is a well-known complication in cancer patients that can lead to prolonged hospital stays. Severe sepsis, defined as a systemic inflammatory response leading to acute organ dysfunction, accounts for 16.4 cases per 1000 people with cancer per year [6]. According to the same study, severe sepsis accounts for 8.5% of all cancer deaths. Angus et al. show that one in every six patients with severe sepsis has underlying malignancies that put them at a 30% higher risk of mortality as compared to other patients with severe sepsis [7].

Common causes of bloodstream infections in neutropenic patients include Gram-negative bacteria such as *Escherichia coli*, *Pseudomonas aeruginosa*, and *Klebsiella pneumoniae*. This is followed by Gram-positive bacteria, most commonly from coagulase-negative *Staphylococcus*. One of the newer and rarer causes of infection is the Gram-negative rod *Elizabethkingia meningosepticum* [8]. There is a rising trend in the number of *E. meningosepticum* infections in hospitalized individuals with severe chronic diseases, especially in the past five years. This suggests that *E. meningosepticum* is emerging as an important pathogen in the immunocompromised population. A recent study noted 118 cases of various presentations of *E. meningosepticum* in Taiwan and suggested a strong connection of the infection with underlying comorbidities like malignancy (36%) and diabetes mellitus (25%) [9].

In another study of 16 patients, *E. meningosepticum* bacteremia was associated with nosocomial infections such as pneumonia, endocarditis, soft tissue infections, osteomyelitis, and catheter-related bacteremia [10]. Only two out of the 16 patients in this study had underlying malignancies. Lab findings were significant for leukocytosis in seven patients and leukopenia in two patients. Some patients were also found to have thrombocytopenia (Platelet <100,000/uL). Only three patients had normal blood counts. In our case series, Case 1 showed leukocytosis (WBC 11,000/mL), and Cases 2 and 3 showed thrombocytopenia (Platelet 26,000/mL) and leukopenia (WBC 2,200/mL) respectively in the setting of *E. meningosepticum* infection; however, this may also be related to their hematological malignancies and chemotherapy and not the ongoing infection (Table 2). It is known that appropriate antimicrobial therapy may be ineffective in resolving *E. meningosepticum* bacteremia if the implanted devices that are colonized are not identified and removed [11]. Disinfection of instruments and medical equipment with 70% alcohol, reducing the use of unnecessary medical devices and invasive procedures, and replacing faulty equipment have been suggested as methods to contain outbreaks [10].

**Table 2:** Summary of case findings from the retrospective chart review AML: acute myelogenous leukemia

	Case 1	Case 2	Case 3
Age (years)	72	50	65
Gender (male/female)	Male	Female	Male
Past Medical History	Metastatic melanoma	Relapsed AML	Esophageal adenocarcinoma (stage IV), AML, pneumonia
Blood Pressure (mmHg)	90/69	118/73	140/70
Heart Rate (bpm)	139	119	110
Body Temperature (°C)	35.9	38.5	-
Isolated Microorganism	Elizabethkingia meningosepticum	*Elizabethkingia meningosepticum*, *Leclercia adecarboxylata*	Elizabethkingia meningosepticum
Pharmacological Treatment	ciprofloxacin, cefoxitin, minocycline, metronidazole	ciprofloxacin, meropenem, minocycline	ciprofloxacin, minocycline

The etiology of sepsis in neutropenic patients has shifted from Gram-positive to Gram-negative bacteremia over the past few decades. Multidrug-resistant, Gram-negative bacilli are becoming increasingly challenging to treat and therefore current guidelines recommend the use of an anti-pseudomonal beta-lactamase with or without an additional antimicrobial as the empiric therapy in Gram-negative bacteremia [12]. *E. meningosepticum* is widely resistant to antibiotics frequently prescribed for Gram-negative bacteremia [13]. Many *Elizabethkingia* spp. possess two different types of β-lactamases: class A extended-spectrum β-lactamases (ESBLs) and class B metallo-β-lactamases (MBLs) rendering them resistant to all β-lactam including carbapenems, aztreonam, tigecycline, and polymyxin [14]. Some patients had a history of multidrug-resistant *Pseudomonas *or *Klebsiella *infection, which was being treated with colistin prior to acquiring an *E. meningosepticum* infection. Prior treatment with colistin had a strong association with the emergence of *E. meningosepticum* bacteremia in these cases owing to their unusual patterns of resistance [15]. High levels of antimicrobial resistance may also be attributed to the patients’ prior history of preceding broad-spectrum antibiotic use, which has been previously shown to be a common factor in infected patients [16].

*E.* *meningosepticum* shows varied degrees of susceptibility to antibiotics used to treat Gram-positive infections including piperacillin-tazobactam, fluoroquinolones, trimethoprim-sulfamethoxazole (TMP-SMX), vancomycin, and rifampicin [17]. This unique nature makes it difficult to choose the appropriate antibiotic therapy. In the 1990s, vancomycin and rifampicin were considered the optimal choice for empiric therapy; however, increased resistance to these drugs has led to decreased usage. More recently, TMP-SMX and fluoroquinolones have shown excellent in vitro activity against *E. meningosepticum* making these the empirical therapy of choice [18]. The superior outcome with fluoroquinolone is attributed to its pharmacodynamics and pharmacokinetic properties along with the ability to maintain optimal cerebrospinal fluid (CSF) levels in vivo [19]. Another study reported that patients with *E.* *meningosepticum* bacteremia had 100% susceptibility to minocycline and 74.4% susceptibility to TMP-SMX and ciprofloxacin [20]. All the patients in our case series showed a good response to a combination of minocycline and ciprofloxacin.

Therefore, a combination therapy is recommended over monotherapy in multidrug-resistant *E. meningosepticum* bacteremia. It has been suggested that prolonged therapy with combinations of rifampin with vancomycin, TMP-SMX, minocycline, or fluoroquinolones may have better clinical outcomes [21]. There is a high 28- day mortality rate (41%) associated with *E. meningosepticum* bacteremia in healthcare-associated bacteremia in adults with underlying conditions [22]. Given the increased risk of mortality and multidrug resistance associated with the organism, it is imperative that clinicians are able to detect and treat the infection appropriately. 

## Conclusions

There is a rising trend of *E. meningosepticum* bacteremia in cancer patients with multiple comorbidities due to prolonged hospitalization and frequent instrumentation. In this report, we present three patients with a hematologic malignancy and neutropenia who developed *E. meningosepticum* infection. Given the challenging clinical course of these patients, it is important to recognize this pathogen as an important cause of bacteremia in immunocompromised patients. Early identification of the pathogen along with empiric treatment with a fluoroquinolone and/or minocycline is imperative to reduce morbidity and mortality.
